# Cell Self-Destruction (Programmed Cell Death), Immunonutrition and Metabolism

**DOI:** 10.3390/biology12070949

**Published:** 2023-07-03

**Authors:** Ligen Yu

**Affiliations:** Talent Recruitment and Career Support (TRACS) Office, Nanyang Technological University, N2.1 B4-01, 76 Nanyang Drive, Singapore 637331, Singapore; mlgyu@ntu.edu.sg

The main purpose of this Special Issue is to provide readers with current understandings of the interactions and causal relations among injury stimuli (including microorganism infections), immune response and overnutrition/lipotoxicity in disease pathogenesis. Special emphasis is placed on microbiota infection, cell self-destruction in response to inflammation, metabolic homeostasis and transient overnutrition in disease initiation, progression and resolution through the dissipation of nutrients for cell metabolism and tissue regeneration.

During the COVID-19 pandemic, which was experienced by the whole world in the past three years, the biggest mystery was the heterogenous and individualized response of the human immune system [1]. Why were most of COVID-19 cases asymptomatic or mild, while some cases were severe or fatal, featured by a cytokine storm? A better understanding of COVID-19 as a disease requires distinguishing between an infection and a disease [2]. Infection is the presence of microorganisms [2], while disease is the symptoms and pathological conditions. Furthermore, disease severity is affected by many factors including pathogen virulence, pathogen load and the inflammatory response by the immune system [2]. In asymptomatic or mild COVID-19 cases, the SARS-CoV-2 viral infection is self-limiting [3,4], and the damaged cells can be effectively cleared by localized programmed cell death via processes like apoptosis, pyroptosis [5] and necroptosis [6]. Yet, in severe cases of COVID-19, of which individuals with metabolic syndromes such as hypertension, type 2 diabetes mellitus, cardiovascular disease, morbid obesity and chronic pulmonary disease were at particularly high risk [3,4], the immunopathological change is distinct [3,4], making severe COVID-19 more of an autoimmune disease [7] than a simple viral infection. Due to the pre-existing overnutrition state in the body, which is worsened by the conversion of nutrients from deceased cells within macrophages, lipotoxicity surpasses SARS-CoV-2 viral infection and becomes the dominant injurious stimulus for cell damage [8,9], which leads to systemic inflammation and multiorgan failure in severe cases [3,4].

Immunologists have long been puzzled by the self-destructive nature of the inflammatory response [10]. In our recent article, we proposed the “self-destroy and rebuild” strategy of inflammatory response [11]. Inflammation can be elicited by various harmful stimuli, such as microbial/viral infections, allergic reactions, chemical insults, lipotoxicity and tissue damage [12]. The process of breaking down damaged cells and converting them into various macronutrients for tissue regeneration and cell metabolism is one of the most important functions of the immune system in maintaining health. A localized self-destructive inflammatory response is protective if the immune system can effectively eliminate the harmful stimuli and initiate the healing process. Cell self-destruction (programmed cell death) includes apoptosis, pyroptosis, necroptosis and necrosis [13]. Phagocytosis is employed to remove various cell debris produced by cell self-destruction and converts these debris into macronutrients. The immune system thus becomes a powerful nutrient regenerator. At this time, nutrient metabolism and immunity are interrelated and integrated and play essential roles in disease prevention and immunonutrition acquisition [14]. In the event of microbial/viral infections, the nutritional flux produced by infected host cell self-destruction (inflammation) together with those produced by daily homeostatic apoptosis [15] may create transient overnutrition [16]. Such transient overnutrition can be dissipated through cell or tissue regeneration. From the above, we can find that as a defense measure, a self-destructive inflammatory response is essential for health and daily homeostasis.

Inflammation becomes problematic only when the injurious stimulus cannot be resolved by a self-destructive inflammatory response and is instead escalated. One such case is lipotoxicity as the injurious stimulus as well as the inflammatory response product. It is well documented that excessive serum amino acids from high protein feeding results in increased hepatic de novo lipogenesis [17,18]. As cell self-destruction and cell debris degradation can also generate amino acids that circulate throughout the circulatory system, when the nutrition generated by the degradation of infection-damaged cells exceeds the nutritional requirements for tissue regeneration, most of the excess nutrients will be converted into lipid intermediates [16]. Lipid intermediates will invade healthy non-adipose tissue, leading to lipotoxicity [8,9] and further tissue damage. Damaged cells are forced to undergo programmed cell death and to produce more nutrients. In such a case, the main product (lipid intermediates) of the inflammatory response is also a strong harmful stimulus for tissue/cell damage and is amplified during the inflammatory response, forming a vicious cycle, making inflammatory response extremely destructive [16].

Owing to the self-destructive inflammatory response and the swift and efficient removal of dead cell debris by efferocytosis [15], most microorganism infections are self-limiting, and inflammation is often asymptomatic, even though these microorganisms also cause harm to human somatic cells. Although for convenience, these “non-invasive” microorganisms are defined as commensal microbes and disease-causing microorganisms are defined as pathogens, no sharp and clear distinction exists between pathogens and symbiotic microbes. On one hand, microbial cells are closely related to human health [19]. Without the support of full-spectrum essential nutrients from diverse microbiota, malnutrition and nutritional imbalances occur, leading to metabolic syndrome, including morbid obesity, diabetes, liver disease, allergies and a compromised immune system [20]. On the other hand, many of these microorganisms in the normal microbiota are opportunistic pathogens [21]. In a state of overnutrition, the efferocytosis process may be delayed, leaving the uncleared self-destructed somatic cell debris as the nutrition base for microorganism proliferation. Overgrowth of any microbial species in the human body (like in the respiratory or gastrointestinal tract) coupled with the lipotoxicity from overnutrition can exacerbate inflammation at these sites and lead to disease [22]. It is the effective programmed cell death pathways running under a balanced nutrition state in the body that shapes a potentially pathogenic microbiome into a commensal microbiome.

Programmed cell death can also happen in a non-inflammatory apoptotic way. Apoptosis occurs in various tissues of all multicellular organisms during development and homeostatic renewal of cells [15]. Dead cells and cell debris must be removed before being replaced to maintain normal functioning of an organ and to avoid extensive inflammatory responses [15]. Problematic clearance of apoptotic cell debris will cause all kinds of diseases [15]. For example, apoptosis resistance in Epstein–Barr virus (EBV)-positive cells is closely associated with nasopharyngeal carcinoma (NPC) [23].

In summary, this Special Issue invites new research articles and reviews on topics related to (but not limited to) microbial/viral-infection-induced host cell self-destruction and immunonutrition acquisition, programmed cell death, metabolites, cell self-destruction in illness, transient overnutrition, lipotoxicity, involuntary weight loss and autoimmunity.
